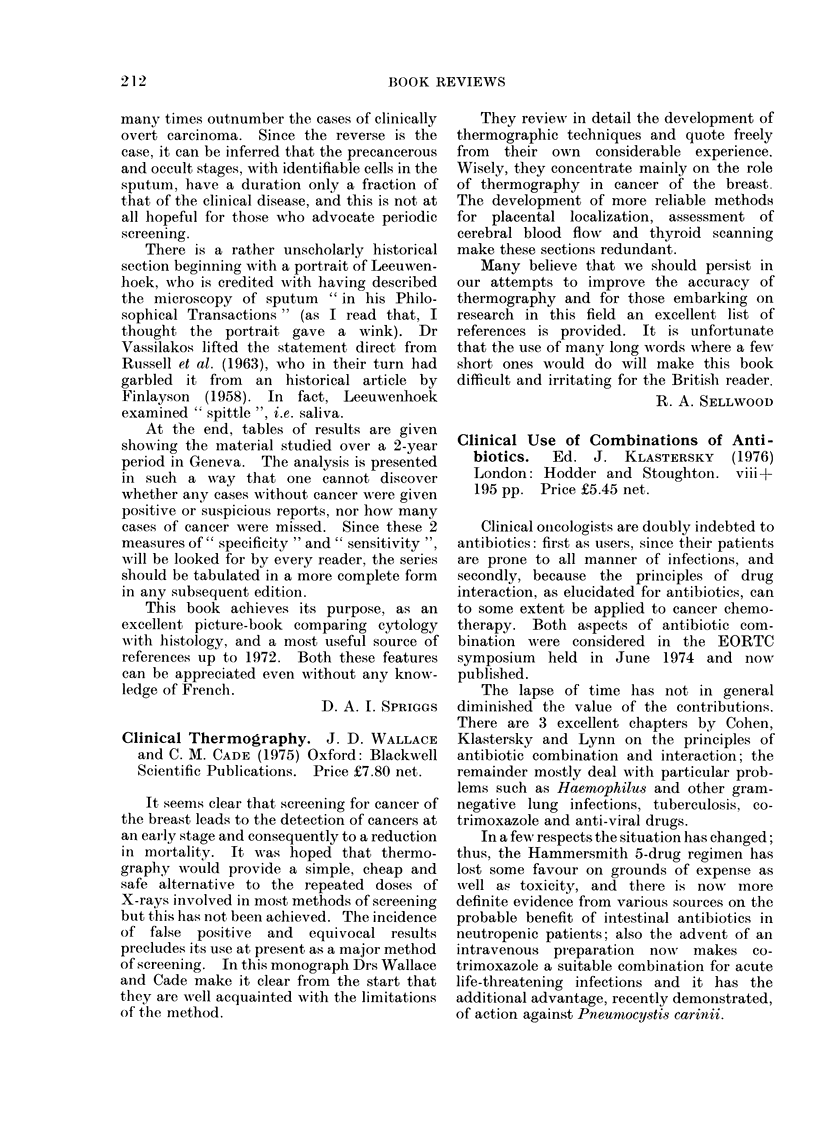# Clinical Thermography

**Published:** 1976-08

**Authors:** R. A. Sellwood


**Clinical Thermography.** J. D. WALLACE and C. M. CADE (1975) Oxford: Blackwell Scientific Publications. Price £7.80 net.

It seems clear that screening for cancer of the breast leads to the detection of cancers at an early stage and consequently to a reduction in mortality. It was hoped that thermography would provide a simple, cheap and safe alternative to the repeated doses of X-rays involved in most methods of screening but this has not been achieved. The incidence of false positive and equivocal results precludes its use at present as a major method of screening. In this monograph Drs Wallace and Cade make it clear from the start that they are well acquainted with the limitations of the method.

They review in detail the development of thermographic techniques and quote freely from their own considerable experience. Wisely, they concentrate mainly on the role of thermography in cancer of the breast. The development of more reliable methods for placental localization, assessment of cerebral blood flow and thyroid scanning make these sections redundant.

Many believe that we should persist in our attempts to improve the accuracy of thermography and for those embarking on research in this field an excellent list of references is provided. It is unfortunate that the use of many long words where a few short ones would do will make this book difficult and irritating for the British reader.

R. A. SELLWOOD